# Depression and Health Risk Behaviors: Towards Optimizing Primary Care Service Strategies for Addressing Risk

**DOI:** 10.4172/2167-1079.1000152

**Published:** 2014-02-14

**Authors:** Joan Rosenbaum Asarnow, Luis Roberto Zeledon, Elizabeth D’Amico, Anne LaBorde, Martin Anderson, Claudia Avina, Talin Arslanian, Minh-Chau Do, Jessica Harwood, Steven Shoptaw

**Affiliations:** 1 UCLA School of Medicine, Los Angeles, California 90095, USA; 2 Kaiser Permanente Los Angeles Medical Center, Los Angeles, USA; 3 RAND, California, USA; 4 Harbor-UCLA Medical Center, Torrance, CA 90502, USA; 5 UC Davis School of Medicine, Sacramento, CA 95817, USA; 6 San Diego State/UCSD, San Diego, CA 92182, USA

**Keywords:** Depression, Health, Sex, Obesity, Smoking, Drug, Marijuana, Alcohol, Primary care

## Abstract

**Purpose:**

Depression and health risk behaviors in adolescents are leading causes of preventable morbidity and mortality. Primary care visits provide prime opportunities to screen and provide preventive services addressing risk behaviors/conditions. This study evaluated the co-occurrence of depression and health risk behaviors (focusing on smoking, drug and alcohol misuse, risky sexual behavior, and obesity-risk) with the goal of informing preventive service strategies.

**Methods:**

Consecutive primary care patients (n=217), ages 13 to 18 years, selected to over-sample for depression, completed a Health Risk Behavior Survey and the Diagnostic Interview Schedule for Children and Adolescents (DISC) depression module.

**Results:**

Youths with DISC-defined past-year depression were significantly more likely to report risk across multiple risk-areas, Wald X^2^(1)=14.39, p<.001, and to have significantly higher rates of past-month smoking, X^2^(1)=5.86, p=.02, substance misuse, X^2^(1)=15.12, p<.001, risky sex, X^2^ (1) =5.04, p=.03, but not obesity-risk, X^2^ (1) =0.19, p=.66. Cross-sectional predictors of risk behaviors across risk areas were similar. Statistically significant predictors across all risk domains included: youths’ expectancies about future risk behavior; attitudes regarding the risk behavior; and risk behaviors in peers/others in their environments.

**Conclusions:**

Depression in adolescents is associated with a cluster of health risk behaviors that likely contribute to the high morbidity and mortality associated with both depression and health risk behaviors. Consistent with the United States National Prevention Strategy (2011) and the focus on integrated behavioral and medical health care, results suggest the value of screening and preventive services using combination strategies that target depression and multiple areas of associated health risk.

## Introduction

Depression is a common problem, associated with significant morbidity and mortality [1]. Primary care is a frequent entry point into the health care system for depressed patients; and most adolescents have access to primary care [1–3]. The United States Preventive Services Task Force recommends primary care screening for depression in adolescents when evaluation and treatment are available(www.uspreventiveservicestaskforce.org/uspstf/uspschdepr.htm) [1]. This recommendation is supported by evidence that providing evidence-based depression care for adolescents through primary care is associated with improved patient outcomes, and consistent with the current emphasis on integrated behavioral and medical health care and Patient Centered Medical Homes [4,5].

Despite the promise of integrated medical and behavioral health care for enhancing access to care, there are a number of barriers to integrating depression and behavioral health care within primary care settings. These include: brief visits, the large number of conditions/behaviors requiring evaluation, provider perceptions of inadequate knowledge; and ambiguity/concerns regarding specialty care linkage. When depression care is offered through primary care, treatment rates are relatively low [4] perhaps related to the general focus on health vs. mental health.

Health risk behaviors (HRBs) such as smoking, substance misuse/abuse, risky sex, and obesity also contribute to adolescent morbidity/mortality and later health problems. Nationwide, tobacco, alcohol and drug abuse are leading preventable causes of death in the United States, [1] nearly half of new STD infections occur among youths ages 15–24 (cdc.gov/healthyyouth/sexualbehaviors), and roughly 28% of youths are overweight/obese. (cdc.gov/healthyyouth/yrbs/pdf/us_obesity_trend_yrbs.pdf). “Problem behavior theory” posits that “problem behavior proneness” is associated with the interaction of personality (attitudes, expectations), perceived environment, and behavior (rates of other problem-behaviors) systems, resulting in the co-occurrence of different HRBs, and is supported by some data indicating associations among HRBs and depression [1,6–11].

This study evaluates the extent to which depression and four major domains of HRBs tend to co-occur, focusing on four common areas of health risk in adolescents which often extend into adulthood contributing to illness and early mortality: smoking, substance use, risky sexual behavior, and obesity risk. This study builds on prior work [7–11] and expands this work by looking at multiple domains of health risk within a primary care sample. Most prior work has used survey samples and/or examined a more limited range of health-risk areas. We also used brief screening measures that can be adapted for primary care screening programs.

Consistent with “problem behavior theory” and the concept of an underlying “problem-behavior proneness,” [6] we predicted that primary care patients with depressive disorders, when compared to less depressed patients, would be significantly more likely to: 1) show a pattern of risk across multiple health-risk domains, and 2) have higher rates of HRBs within each risk-domain. To further inform preventive service strategies, we also conducted exploratory analyses focusing on each area of health-risk and examining whether similar attitudes and social/environmental influences contributed to risk across health risk-domains. If depression and HRBs co-occur and are associated with similar risk-processes, preventive services employing combination strategies that simultaneously target multiple HRBs and risk-processes are likely to be more effective than preventive service strategies that focus on a single risk-area.

## Methods

This is the first paper on the 24-7 HEALTH study, a study examining depression and health risk behaviors among primary care patients from two diverse health care organizations purposely selected to include one managed care organization and a medical center accepting a variety of insurance plans. The study was approved by the institutional review boards from both participating organizations. Informed consent was obtained from all parents for youths <18 years, with informed assent from youth; and all youths over 18 years of age. This study reports on results from the baseline assessment. Future publications will focus on the intervention phase of the project.

Consecutive patients, ages 13–18 years inclusive, were invited to participate in the study. To oversample for depression, patients were asked to complete a 4-item depression screener while waiting for their appointments. Screener items included: 1) two Composite International Diagnostic Interview (CIDI) past-year depression items assessing dysphoric mood and anhedonia (as modified for adolescents in prior research); [4] and 2) two parallel items asking about dysphoria and anhedonia “often during the past month”. Positive screens on any item resulted in study eligibility, with exclusions for functioning/characteristics that would interfere with study procedures: lives over 1 hour away from site; youth not English-speaking; parents not English or Spanish-speaking.

Screening involved anonymous voluntary questionnaires with no identifiable information, and was done with a waiver of parent informed consent, and most youths (88.7%) agreed to complete the anonymous screener (1324/1493), To enroll in the study, youths had to agree to allow contact with parents for informed consent and be willing to return to the clinic for assessments and the study intervention. Of 491 eligible participants, 279 completed informed consent/assent procedures with youths and parents, and 217 completed the study assessments in an additional clinic-visit. Enrollment occurred between December 2007 and November 2010.

## Measures and Procedures

*The NIMH Diagnostic Interview Schedule for Children (DISC-IV) depression module*, a structured diagnostic interview with established inter-rater and test-retest reliability, was administered by trained interviewers using a computer assisted format [12]. Interviewers were trained, certified, and supervised by a senior staff member trained by the DISC development team. Quality assurance ratings completed on 20% of interviews (randomly selected) indicated strong quality (Mean=1.2, SD=0.54, 3-point scale 1=good to 3=poor).

Due to the sensitive nature of HRB questions, the *HRB Survey* was completed individually by youths at a private computer station, with assistance available if requested. The HRB Survey used items from the Youth Risk Behavior Survey [13], National Longitudinal Study of Adolescent Health [14], Monitoring the Future [15] and other national surveys [16,17]. Questions asked about: past-month smoking and substance use (alcohol, marijuana, other drugs) and attempts to cut down/quit [15,16]; sex without a condom, number of partners during the prior 6-months, Sexually Transmitted Infections/Diseases (STI/STD), and pregnancy [13,16]. *Substance use-related impairment was assessed using the Substance Use Scale of the Problem Oriented Screening Instrument for Teenagers (POSIT)* [18]. *The Body Mass Index (BMI),* based on objective measurement of height and weight by the study assessor, adjusted for sex and age (http://apps.nccd.cdc.gov/dnpabmi/), indexed obesity-risk. We also asked about: 1) expectancies that youth would have the “*HRB*” in 6-months?” [16,19] 2) attitudes regarding the health risk behavior [16,17,20]; 3) resistance self-efficacy [14,16,19]; 4) perceptions that peers (students in your grade) would have the “*risk-behavior*”?[ 19,20]; 5) peer and parent distress if youth engaged in the risk-behavior [16]; and 6) whether youth’s best friend, parents, or others they “hung around with” had the risk-behavior [19,21]. Parallel scales assessed variables related to diet, exercise, risky sex, and sex.

*A composite HRBI* was derived based on the sum of four binary (0/1) HRB indicators: 1) past-month smoking/tobacco use, 2) substance misuse, defined as past-month alcohol use with impairment (POSIT ≥ 1) or illegal drug use (marijuana or other drugs), 3) unprotected sex (without condom) in the past 6-months; 4) obesity risk, defined as BMI-for-age-gender ≥ 85th percentile (http://www.win.niddk.nih.gov/statistics/index.htm). These four risk-indicators also served as risk-indicators for their respective risk-domains.

## Statistical Analyses

We present standard descriptive statistics, examined the effects of past-year depressive disorder on the HRBs using logistic regression analyses predicting to dichotomous variables and negative binomial regression analyses predicting to dimensional variables (HRBI, POSIT). Because preliminary analyses indicated that depression was more common in girls (X^2^ (1)=11.29, p<.001) and age and Hispanic ethnicity were significantly associated with the HRB measures, analyses adjusted for gender, age, and Hispanic ethnicity. Because we aimed to evaluate overall depression effects and data indicate that subsyndromal depression is associated with increased risk of depressive disorder-onset and impairment levels comparable to those for depressive disorder [22], we collapsed across these categories and compared youths with no depressive disorder to those with disorder broadly-defined to include DISC-definite and intermediate diagnoses. Youths with definite and intermediate diagnoses did not differ significantly on any of the HRB measures and results were similar, with no change in conclusions, when these subgroups were examined separately and when depression was entered as a 3-level variable. Next, we examined the effects of the attitude and social-environmental variables on the binary HRBs using logistic regression. These analyses clarify bivariate associations among the binary HRBs and the predictor variables adjusting for control variables (gender, age, Hispanic ethnicity). Finally, to identify the most parsimonious set of predictors, we used logistic regression with a backwards stepping procedure including all terms significant in the initial analyses and depressive disorder in the model, with variables removed based on their p-values in a descending order and retaining only variables with p-values ≤ 0.10. Statistical analyses were conducted using SPSS, Version 20, except SAS PROC GENMOD was used for negative binomial regression analyses.

## Results

The sample included 119 females (55% of sample, mean age=16.01, SD=1.45), and 98 males (45% of sample, mean age=15.90, SD 1.55). Participants were ethnically diverse: 70% endorsed Hispanic/Latino ethnicity, 8% African-American, 3% Asian, 2% Alaskan Native/American Indian, 12% mixed race, and 12% Caucasian. Roughly half of the sample (98/217, 45%) met DISC-criteria for past-year definite (n=51) or intermediate depressive disorder (n=47).

### Distribution of risk within the sample

As shown in (Table 1), the HRB domain with the most at-risk youths was obesity. BMI measurements fell in the obese/risk-for-obesity range for 50.2% of youths, 31.3% classified as obese. Substance misuse was the next most common risk-domain, followed by risky sex, and past-month smoking. Smoking was associated with significantly higher rates of: substance misuse, X^2^(1) =45.93, p<.001; and risky sex, X^2^(1) =21.00, p<.001; and substance misuse was associated with significantly higher rates of risky sex, X^2^(1) =26.74, p<.001. Associations between obesity-risk and the other HRBs were not statistically significant: smoking, X^2^(1)=0.34, p=.56; substance misuse, X^2^(1) =0.40, p=.53; risky sex, X^2^(1)=2.90, p=.09. Multiple risk indicators were present in 33.6% of the sample: 2 risk areas (20.7%); 3 risk areas (10.1%), four risk areas (2.8%); risk in ≥ 1 area (73.3%).

### Depression and HRBs

**Figure 1:**
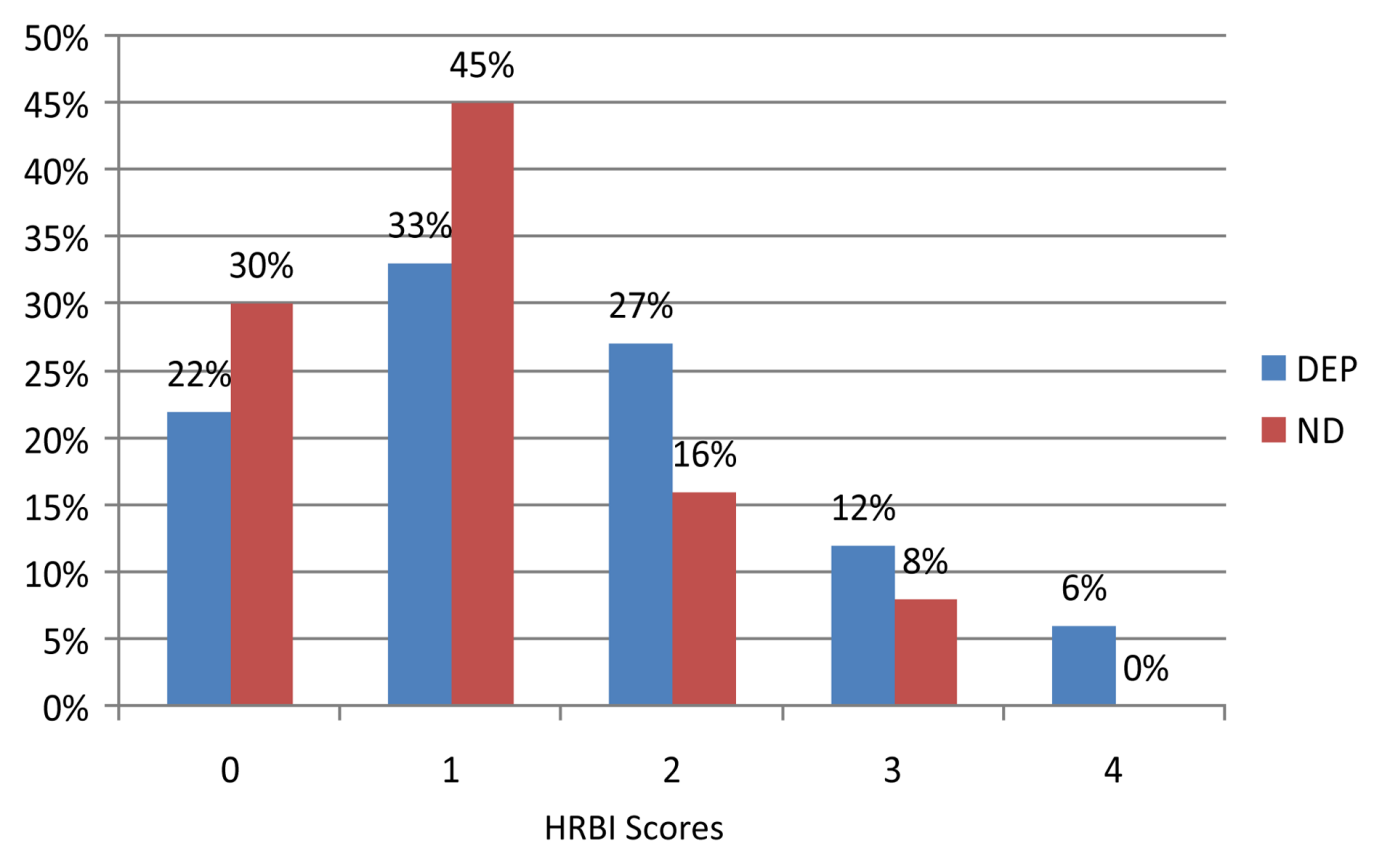
Health Risk Behavior Index (HRBI) Scores for Depressed and Non-Depressed Youths.

As predicted, depressive disorder status was a significant predictor of the HRBI, sum of risk levels across the four targeted risk areas (Table 2). Youths with depressive disorder were significantly more likely to report risk across multiple domains, with 45% of depressed youths showing risk in two or more domains vs. 25% of non-depressed youths (Table 2). All youths with risk across all four risk-domains suffered from past-year depression (Figure 1).

Depressive disorder status was significantly associated with most domain-specific risk indicators, specifically: past-month smoking; substance misuse; problematic drug use (drug use with impairment); problematic alcohol use, defined as alcohol use with impairment; substance use-related impairment on the POSIT; risky sexual behavior (without condom) during the preceding 6-months; more sexual partners within the past 3-months; and marginally associated with increased rates of STI/STDs (Table 2). Rates of obesity were similar across depressed and non-depressed groups.

### Descriptive and exploratory analyses of risk in each health-risk domain

#### Smoking

Over half the sample (64.1%) reported never having smoked; 35 youths reported past-month smoking, 25 of whom (71.4%) reported ≥ 1 quit attempt. Table 3 presents results of the logistic regression analyses examining associations among the binary HRBs (e.g. post-month smoking) and each attitude and social-environmental variable. These analyses revealed that smoking was significantly associated with: expecting to smoke during the next 6-months; lower resistance self-efficacy/perceived ability to resist smoking; more positive attitudes towards smoking; the amount of time the youth spent with individuals who smoke and perceptions that parents would be upset if youth smoked. When all significant variables and depressive disorder status were entered in the backwards stepping procedure, results indicating a statistically significant effect only for expecting to smoke during the next 6-months, OR 0.36, 95% CI 0.20, 0.68, X^2^=10.23, p<.001, supporting the strength of the expectancy variable as a cross-sectional predictor. However, marginal effects for other variables suggest that non-overlapping variance from other variables also contributed to prediction: resistance self-efficacy, OR 0.85, 95% CI 0.70, 1.02, X^2^=3.07, p<.08; attitudes towards smoking, OR 1.15, 95% CI 0.98, 1.35, X^2^=2.91, p<.09; hanging around with other smokers, OR 0.61, 95% CI 0.35, 1.06, X^2^=3.08, p<.08, and older age, OR 1.41, 95% CI 0.98, 2.02, X^2^=3.44, p<.06.

#### Alcohol use

Lifetime drinking was reported by 141 youths (65%), 44 (20.3%) reported current drinking with impairment, 22 of whom (20.3%) reported past-month efforts to quit drinking. Statistically significant predictors of problematic drinking included: expecting to drink during the next 6-months; low resistance self-efficacy, more positive attitudes towards drinking; perceptions that peers drank alcohol; having a best friend who drank; hanging around with people who drank; expected that peers would be upset if they found out they drank alcohol; and expected that their parents would be upset if they found out they drank alcohol (Table 3). The final model when depression and all statistically significant variables from the bivariate analyses were included in the model yielded significant effects for expecting to drink, OR 0.51, 95% CI 0.31, 0.83, X^2^=7.24, p<.007;resistance self-efficacy, OR 0.83, 95% CI 0.72, 0.96, X^2^=6.38, p<.012; and marginal effects for drinking attitudes, OR 1.10, 95% CI 0.99,1.22, X^2^=3.37, p<.07; perceptions that peers drank, OR 0.1.18, 95% CI 0.99, 1.42, X^2^=3.37, p<.067; male gender, OR 2.17, 95% CI 0.92, 5.14, X^2^=3.12, p<.08; and non-Hispanic ethnicity, OR 2.36, 95% CI 0.91, 6.14, X^2^=3.09, p<.08.

#### Drug use

Ninety-three youths (42.9%) reported lifetime marijuana use; 48 reported past-month use, 29 of whom (60.4%) reported ≥ 1 quit attempt. Other drug use was less common (amphetamines/uppers n=11, 5.1%; ecstasy n=10, 4.6%; hallucinogens n=3, 1.4%) and generally occurred with marijuana use (n=5 past-month marijuana use only, n=32 marijuana and other drugs, X^2^(1)=39.46, p<.001). Marijuana use was significantly associated with: expecting to use marijuana during next 6 months; low resistance self-efficacy; more positive attitudes towards marijuana; perceptions that peers used marijuana; having a best friend who used marijuana, hanging around with people who used marijuana; and expecting low levels of peer and parent upset if used (Table 3). The final model yielded significant effects for attitudes towards marijuana, OR 1.29, 95% CI 1.13, 1.47, X^2^=13.58, p<.001; resistance self-efficacy, OR 0.84, 95% CI 0.72, 0.98, X^2^=5.07, p<.03; hanging out with other marijuana-users, OR 0.35, 95% CI 0.17, 0.70, X^2^=8.56, p<.003; expected peer distress if used, OR 2.29, 95% CI 1.14, 4.60, X^2^=5.43, p<.02; and a marginal effect for expecting to use, OR 0.57, 95% CI 0.31, 1.05, X^2^=3.25, p<.07.

#### Sexual behavior

Eighty-nine youths (40.8%) reported having engaged in sexual intercourse. Fifteen youths (8 girls, 7 boys) endorsed prior pregnancies/getting someone pregnant, with 3 youths reporting >1 pregnancy and one an abortion, and 19 youths (8.8%) reporting STI/STDs. Risky sex (without condoms) was significantly associated with: expecting to have risky sex during next 6 months; more liberal attitudes towards sex; perceptions that peers were engaging in risky sex; perceptions that peers were engaging in sex, OR 1.25, 95% CI 1.10, 1.41, X^2^=12.33, p<.01; perceptions that peers had more liberal attitudes towards sex, OR 0.54, 95% CI 0.36, 0.81, X^2^=9.16, p<.01; and marginally associated with family attitudes supporting safe sex, OR 2.09, 95% CI 0.93, 4.71, X^2^=3.18, p=.08 (Table 3). The final model included significant effects for: expecting to have risky sex, OR 0.23, 95% CI 0.14, 0.36, X^2^=38.09, p<.001; perceptions that peers were engaging in sex, OR 1.28, 95% CI 1.08, 1.51, X^2^=8.02, p<.005; and older age, OR 1.55, 95% CI 1.15, 2.07, X^2^=8.46, p<.004; and a marginal effect for female gender, p<.07.

#### Obesity-risk

On self-report, 77% of youths (n=167) endorsed having felt overweight in their lifetimes, 122 youths (56.2% of sample) reported current efforts to lose weight. During the past-month, 124 youths (57.1%) reported exercising to lose weight; 91 (41.9%) reported dieting to lose weight. Youths with obesity-risk (objectively-measured BMI) were significantly more likely to report: trying to lose weight (X^2^(3)=83.68, p<.001), dieting to lose weight (X^2^(1) =17.70, p<.001), and exercising to lose weight (X^2^(1)=18.59, p<.001).

Significant predictors of obesity-risk included: expecting to be overweight; diet self-efficacy; diet attitudes; having an overweight best friend; having overweight parents and siblings; and “hanging out” with overweight people (Table 3). Obesity-risk was not significantly associated with beliefs supporting exercise, self-efficacy for exercise, or perceptions that peers are overweight. The final model included significant effects for expecting to lose weight, OR 0.30, 95% CI 0.21, 0.43, X^2^=42.63, p<.001; having an overweight best friend, OR 3.25, 95% CI 1.51, 7.02, X^2^=9.05, p<.003; diet attitudes, OR 1.09, 95% CI 1.01, 1.18, X^2^=4.81, p<.03; and a marginal trend for obesity risk to be associated with younger age, OR 0.81, 95% CI 0.64, 1.02, X^2^=3.16, p<.08.

## Conclusions

Consistent with the United States National Prevention Strategy [1], our results support the importance of screening for HRBs. Rates of HRBs were high, with 74% of patients showing risk in ≥ 1 risk-domains. However, as predicted, rates of HRBs were significantly higher among depressed youths and depressed youths were more likely to show multiple areas of health-risk. These data extend prior findings and suggest that elevated levels and patterns of HRBs contribute to the high economic and social costs of depression [7–11]. Depression treatment and course is often complicated by HRBs and extent data indicate that improved depression is associated with reduced HRBs, particularly substance misuse [23–25], underscoring both the need for preventive services targeting risk-reduction in depressed youths and the potential benefits of depression treatment for reducing HRBs.

As predicted and consistent with problem behavior theory [6], some of the most pernicious HRBs clustered together (smoking, substance misuse, risky-sex). For instance, among current smokers in our sample, 80% reported substance misuse, 60% reported engaging in risky sex, and 34% endorsed more than one HRB. These data are consistent with the view that smoking may often be a “gateway” to more extensive drug use and other HRBs and emphasize the potential benefits of intervention strategies that address multiple risk behaviors and the connections between them [6].

Also consistent with problem behavior, social learning, and cognitive-behavioral theory, youths’ expectancies that they would engage in the risk-behavior during the next 6-months were significant cross-sectional predictors of the risk-behaviors. This held for all examined risk areas: smoking, substance misuse (alcohol and marijuana), risky sex, and obesity-risk. Attitudes supporting the risk behavior were also significant predictors of the risk behaviors, although attitudes towards diet but not exercise were significant predictors of obesity-risk, and obesity-risk was associated with greater awareness of the benefits of “healthy diet.” Lower resistance self-efficacy (perceived difficulty resisting the risk behavior) was significantly associated with smoking, alcohol, marijuana use, and obesity-risk.

Supporting the importance of social/environmental influences, some indicator that peers or others in the environment were engaging in the risk behavior was significantly associated with risk in each of the risk-domains. Youths who smoked were significantly more likely to hang out with other smokers; youths with problematic alcohol and marijuana use were significantly more likely to hang out with others who used these substances and to believe that peers would not be upset if they drank or smoked marijuana; and youths who were overweight were more likely to hang out with others who were overweight, have family members who were overweight, and have a best friend who was overweight. Youths who engaged in risky sex believed that a higher proportion of their peers were having sex and having sex without condoms. Again, compatible with problem behavior theory [6], these data suggest that similar kinds of expectancies, attitudes, and environmental factors contribute to risk across the four risk-domains.

Despite the importance of the peer group during adolescence, when examining substance use and smoking, youths who felt their parents would be upset by smoking or substance misuse were significantly less likely to engage in the risk-behavior. These data support the role that parental values and reactions may play in reducing substance use risk, are consistent with data showing the power of family-based preventive strategies [24], and underscore the important influences of both peers and parents on substance use risk.

Our finding that only a subgroup of youths engaging in risk-behaviors had attempted to quit/reduce the risk-behaviors underscores the challenges for risk-reduction. Quit attempts were reported by 50%, 60.4%, and 71.4% of youths with past-month problematic drinking, marijuana use, and smoking respectively. This finding is consistent with literature showing that youth will make self-change efforts and emphasizes the role that motivational strategies can play in reducing HRBs. California, the state where the study was conducted, has strict laws controlling smoking, economic incentives, a stop-smoking advertising campaign, and a past-month smoking rate which is roughly half of the national average, 6.9% vs. 15.9% (ages 12–17) (www.cdc.gov/tobacco/data_statistics/state_data/state_highlights/2010/states/california/index.htm). These data and the high rate of quit attempts observed among smokers in this study suggests that these efforts may be increasing motivation to quit.

In contrast, weight reduction appears to be viewed as necessary by a broad group of youths, with weight loss attempts reported by 85.3% of youths with obesity-risk and 44.4% of youths with lower weights. These data suggest that weight loss attempts may play a role in maintaining healthy weight and are often unsuccessful, given the high-rate of weight loss efforts among youths with obesity-risk.

Study limitations include the focus on cross-sectional predictors; future research is needed to examine whether these cross-sectional predictors will predict longitudinal patterns. While strengths of the manuscript include the fact that we sampled patients from two diverse health care organizations, and oversampling for depression allowed us to obtain a large enough sample of youths with depression to address the study aims, results may not generalize to other samples from other health care facilities and using other sampling strategies. Although comparable to similar studies with waivers for parental consent for screening but not broader study participation, response rates were moderate [4]. The sample was recruited to over-select for depression and focused on depression and four areas of health-risk; other research is needed to examine other health-risk and mental health problems. Youths were recruited from consecutive primary care patients and presented at the clinics with a variety of health conditions, as well as for regular check-ups. Future research is needed to evaluate the impact of other health conditions that lead youths to visit health facilities.

## Implications and Contributions

Risk behaviors are a leading cause of preventable morbidity and mortality and depression is both a leading cause of disability worldwide and associated with suicide, the third leading cause of death in adolescents and young adults [1]. Study results indicate that depression and HRBs (particularly the cluster of smoking, substance misuse, and risky sex) frequently co-occur and risk across diverse risk-domains was associated with similar constructs. These findings support the value of screening and clinical service strategies that target multiple related risk-conditions, the potential benefits of treatment approaches with flexibility to address both depression and HRBs using clinical decision-making algorithms and/or a modular approach [4], and the value of pediatric collaborative care models for integrating behavioral health within primary care. Such combination strategies that maximize the value of primary care encounters for promoting health and reducing risk are consistent with current guidelines and recommendations and are consistent with our national goal of increasing the quality and years of healthy life [1–3,25–27].

## Figures and Tables

**Table 1:** Risk Across Domains of Health Risk Behavior*.

	Total Sample (N=217)		Smoking (N=35)		Substance Misuse (N=68)		Risky Sex (N=61)		Obesity Risk (N=109)	
	f	%	f	%	f	%	f	%	f	%
**Smoking**	35	16.1%			28	41.2%***	21	34.4%***	16	14.7%
**Substance Misuse**	76	31.9%	28	80%***			35	57.4%***	32	29.4%
**Risky Sex**	61	28.1%%	21	60%***	35	51.5%***			25	22.9%
**Obesity Risk**	109	50.2%	16	45.7%	35	46.1%	25	41%		

* Percentages calculated as percent of youths with HRB in column with co-occurring HRB in row.

**Table 2:** Health Risk Behaviors Among Youths with and Without Past-Year Depression.

Variables	NOT DEPRESSED (N=119)		DEPRESSED (N=98)		OR or IRR*	95% CI	X^2^	p
	f or M	% or SD	f or M	% or SD				
**HRBI**	1.03	0.90	1.47	1.15	1.5	1.2, 1.9	14.81	.01
HRBI ≥ 2	29	24.4%	44	44.9%	3.6	1.9, 7.0	14.39	.01
**Smoking, Past Month**	13	10.9%	22	22.4%	2.7	1.2, 6.0	5.86	.02
**Sub Misuse, Past Month**								
Any Problematic Use	26	21.8%	42	42.9%	3.7	1.9, 7.1	15.12	.01
Problematic Alcohol	19	16.0%	25	25.5%	2.3	1.1, 4.7	4.88	.03
Illegal Drug Use	12	10.1%	29	29.6%	5.0	2.3, 11.1	15.91	.01
Impairment, POSIT	1.11 0	0.22 0–11	1.93 0	2.89 0–12	2.0	1.1, 3.6	5.84	.02
**Risky Sex**								
No Condom, 6-Mos	27	22.7%	34	34.7%	2.2	1.1, 4.3	5.04	.03
STI/STDs	7	6.4%	12	14.3%	2.5	0.9, 6.9	3.26	.07
#Partners, 3-Mos.	0.37 0	0.64 0–4	0.56 0	0.85 0–5	1.7	1.1, 2.6	6.86	.01
**Obesity-Risk, BMI**	59	49.6%	50	51.0%	1.1	0.6, 2.0	0.19	.66

* Odds Ratios (OR) reported for logistic regression analyses, incident rate ratios (IRRs) reported for negative binomial regressions which were done for non-categorical variables (HRBI, POSIT, # Partners).

**Table 3:** Cross-Sectional Predictors of Health Risk Behaviors.

	Smoking				Alcohol				Marijuana				Sexual Behavior				Obesity			
Predictor Variables	OR	95% CI	X^2^	p	OR	95% CI	X^2^	p	OR	95% CI	X^2^	p	OR	95% CI	X^2^	p	OR	95% CI	X^2^	p
**Expect Risk Behavior Next 6 Months Smoking**	0.19	0.11, 0.31	41.52	0.01	0.28	0.19, 0.43	35.39	0.01	0.15	0.09, 0.25	51.42	0.01	0.23	0.15, 0.36	39.26	0.01^1^	0.27	0.19, 0.38	53.80	0.01
**Resistance Self-Efficacy**	0.63	0.55, 0.74	36.39	0.01	0.70	0.62, 0.78	35.37	0.01	0.66	0.58, 0.74	51.48	0.01					1.07	1.01, 1.13	5.04	0.03
**Attitudes**	1.36	1.20, 1.54	23.30	0.01	1.27	1.16, 1.38	27.00	0.01	1.38	1.25, 1.53	38.98	0.01	0.52	0.36, 0.77	10.95	0.01	1.08	1.03, 1.12	10.41	0.01
**% Peers Risk Behavior**	1.10	0.95, 1.27	1.67	0.20	1.36	1.17, 1.59	15.90	0.01	1.50	1.29, 1.76	26.00	0.01	1.26	1.09, 1.45	9.94	0.01^1^	1.08	0.95, 1.22	1.41	0.24
**Best Friend Risk Behavior**	2.17	0.97, 4.84	3.59	0.06	5.18	2.30, 11.64	15.84	0.01	6.83	3.26, 14.28	25.99	0.01					3.86	2.02, 7.37	16.71	0.01
**Family Risk Behavior**	1.64	0.77, 3.51	1.61	0.20	1.18	0.43, 3.22	0.10	0.75	2.11	0.73, 6.09	1.89	2.11					2.46	1.40, 4.32	9.89	0.01
**Time with Others Risk Behavior**	0.45	0.29, 0.68	13.85	0.01	0.39	0.26, 0.60	19.02	0.01	0.20	0.12, 0.33	39.85	0.01					0.61	0.43, 0.85	8.53	0.01
**Expected Peer Distress**	0.66	0.39, 1.11	2.48	0.12	0.52	0.35, 0.77	10.47	0.01	0.50	0.34, 0.72	13.79	0.01					0.99	0.68, 1.15	0.00	0.98
**Expected Parent Distress**	0.46	0.27, 0.78	8.21	0.01	0.58	0.39, 0.85	7.60	0.01	0.41	0.25, 0.68	12.09	0.01					1.28	0.95, 1.72	2.66	0.10

1 Question asks about risky sex, defined as sex without a condom

## References

[1] U.S. Department of Health and Human Services, Office of the Surgeon General, U.S. Department of Health and Human Services (2011). National Prevention Strategy.

[2] Cheung AH, Zuckerbrot RA, Jensen PS, Ghalib K, Laraque D (2007). Guidelines for Adolescent Depression in Primary Care (GLAD-PC): II. Treatment and ongoing management. Pediatrics.

[3] Wren FJ, Foy JM, Ibeziako PI (2012). Primary care management of child & adolescent depressive disorders. Child AdolescPsychiatrClin N Am.

[4] Asarnow JR, Jaycox LH, Duan N, LaBorde AP, Rea MM (2005). Effectiveness of a quality improvement intervention for adolescent depression in primary care clinics: a randomized controlled trial. JAMA.

[5] Richardson L, McCauley E, Katon W (2009). Collaborative care for adolescent depression: a pilot study. Gen Hosp Psychiatry.

[6] Jessor R, Chase JA, Donovan JE (1980). Psychosocial correlates of marijuana use and problem drinking in a national sample of adolescents. Am J Public Health.

[7] Katon W, Richardson L, Russo J, McCarty CA, Rockhill C (2010). Depressive symptoms in adolescence: the association with multiple health risk behaviors. Gen Hosp Psychiatry.

[8] Paxton RJ, Valois RF, Watkins KW, Huebner ES, Drane JW (2007). Associations between depressed mood and clusters of health risk behaviors. Am J Health Behav.

[9] Hallfors DD, Waller MW, Ford CA, Halpern CT, Brodish PH (2004). Adolescent depression and suicide risk: association with sex and drug behavior. Am J Prev Med.

[10] Shrier LA, Harris SK, Kurland M, Knight JR (2003). Substance use problems and associated psychiatric symptoms among adolescents in primary care. Pediatrics.

[11] Shrier LA, Harris SK, Sternberg M, Beardslee WR (2001). Associations of depression, self-esteem, and substance use with sexual risk among adolescents. Prev Med.

[12] Shaffer D, Fisher P, Lucas CP, Dulcan MK, Schwab-Stone ME (2000). NIMH Diagnostic Interview Schedule for Children Version IV (NIMH DISC-IV): description, differences from previous versions, and reliability of some common diagnoses. J Am Acad Child Adolesc Psychiatry.

[13] Brener ND, Kann L, Kinchen SA, Grunbaum JA, Whalen L (2004). Methodology of the youth risk behavior surveillance system. MMWR Recomm Rep.

[14] Harris KM, Florey F, Tabor J, Bearman PS, Jones J (2003). The National Longitudinal Study of Adolescent Health: Research and design. WWW document.

[15] Johnston LD, O’Malley PM, Bachman JG, Schulenberg JE (2007). Monitoring the future national survey results on drug use, 1975–2006. Volume I: Secondary school students (NIH Publication No. 07-6205).

[16] Ghosh-Dastidar B, Longshore DL, Ellickson PL, McCaffrey DF (2004). Modifying pro-drug risk factors in adolescents: results from project ALERT. Health EducBehav.

[17] Bogart LM, Elliott MN, Uyeda K, Hawes-Dawson J, Klein DJ (2011). Preliminary healthy eating outcomes of SNaX, a pilot community-based intervention for adolescents. J Adolesc Health.

[18] Rahdert E, U.S. Department of Health and Human Services (1991). The Adolescent Assessment/Referral System Manual.

[19] Stern SA, Meredith LS, Gholson J, Gore P, D’Amico EJ (2007). Project CHAT: a brief motivational substance abuse intervention for teens in primary care. J Subst Abuse Treat.

[20] Van Haitsma M, Paik A, Laumann EO, Ellingson S, Mahay J (2004). The sexual organization of the city.

[21] Colby SM, Monti PM, O’Leary Tevyaw T, Barnett NP, Spirito A (2005). Brief motivational intervention for adolescent smokers in medical settings. Addict Behav.

[22] Gotlib IH, Lewinsohn PM, Seeley JR (1995). Symptoms versus a diagnosis of depression: differences in psychosocial functioning. J Consult Clin Psychol.

[23] McKowen JW, Tompson MC, Brown TA, Asarnow JR (2013). Longitudinal associations between depression and problematic substance use in the Youth Partners in Care study. J Clin Child AdolescPsychol.

[24] Bauman KE, Ennett ST, Foshee VA, Pemberton M, King TS (2002). Influence of a family program on adolescent smoking and drinking prevalence. Prev Sci.

[25] Irwin CE, Adams SH, Park MJ, Newacheck PW (2009). Preventive care for adolescents: few get visits and fewer get services. Pediatrics.

[26] Curry J, Silva S, Rohde P, Ginsburg G, Kennard B (2012). Onset of alcohol or substance use disorders following treatment for adolescent depression. J Consult Clin Psychol.

[27] Goldstein BI, Shamseddeen W, Spirito A, Emslie G, Clarke G (2009). Substance use and the treatment of resistant depression in adolescents. J Am Acad Child Adolesc Psychiatry.

